# News and Coming Events

**Published:** 1907-05-25

**Authors:** 


### NEWS AND COMING EVENTS.

The new South Eastern Hospital, erected to the plans of Messrs. T. W. Aldwinckle and Sons, architects, London, has been ventilated on the "Boyle" natural system.

Sir William Job Collins, F.R.C.S., M.P., will preside at the annual meeting of the Asylum Workers' Association, to be held at the Medical Society's House, 11 Chandos Street, on Wednesday, May 29, at 4 p.m.

The Right Hon. The Lord Mayor will take the chair at the Festival Dinner to be held at the Trocadéro Restaurant on Tuesday, June 4th, at 7.30 p.m., in aid of the City of London Hospital for Diseases of the Chest, Victoria Park, E.

The Bill promoted to obtain the incorporation of King Edward's Hospital Fund for London was before the Examiners of Standing Orders in Parliament this week. After formal proof of compliance with the Standing Orders had been given it was ordered to be reported for second reading in the House of Commons. The measure has already passed through all stages in the Upper House.

At the annual meeting of the North-Eastern Hospital for Children, Hackney Road, Bethnal Green, held on Thursday, May 16, the report showed that the total ordinary expenditure had been £10,927 and the total income £10,083. The chairman stated that since the close of the year the debt resulting from the erection of new buildings had been reduced from £10,500 to £7,500, owing to a generous donation received for the purpose. The great event of last year had been the opening of the nurses' home and the laundry. These buildings had greatly reduced the cost per in-patient per week. It had been decided to make a special appeal for funds. The Lord Mayor had very kindly arranged to preside on May 28 at a banquet at the Mansion House to inaugurate the appeal. It is a remarkable fact that this hospital, with an expenditure of nearly £11,000 a year, has an endowed income of under £250, and that after reckoning the sum arising from patients it is dependent upon voluntary contributions to the extent of no less than £10,000 a year.

The death is announced of Sir Joseph Fayrer, K.C.S.I., LL.D., M.D., F.R.S., Physician Extraordinary to the King since 1901. Sir Joseph, who first started as an engineering student, was born in 1824, and entered Charing Cross Hospital School of Medicine as a student in 1844. Three years later he qualified, and was appointed medical officer of H.M.S. *Victory*. Later he proceeded to Rome, where he took the degree of M.D. In 1850 he entered the Bengal Medical Service, and saw service in Burmah and during the Mutiny. He was in Lucknow during the heroic defence of the city against the rebels, and during the siege he transformed his house into a small fortress and hospital combined. Here, too, he was wounded while operating under fire. For his services he was mentioned in despatches and received promotion. After the Mutiny he went to Edinburgh, where he took his M.D. degree. Returning to India he became Professor at the Calcutta Medical College and President of the Medical Board. The latter post he held till 1895, when he retired, receiving a baronetcy the following year. His contributions to medical and general literature have been numerous, and up to his death he continued to take an active interest in his profession. He was a corresponding member of many learned foreign societies, a Governor of Guy's Hospital, and a Governor and Consulting Physician of the Italian Hospital, London.

The new out-patients' department of the Evelina Hospital for Sick Children, Southwark Bridge Road, S.E., was opened on Thursday, May 23, by the Lord Mayor and the Lady Mayoress.

A Bill is to be introduced by a medical member of the State Legislature in Alabama prohibiting the sale of cocaine and restricting the manufacture of patent medicines in that State.

The second International Congress of Physicotherapy will be held in Rome from October 13 to 17 next under the presidency of Professor Guido Bacelli. The honorary secretary of the Congress is Professor Carlo Colombo, Via Plinia 1, Rome.

The Normanton and District Joint Isolation Hospital has been opened. The site cost £1,700, and £15,500 has been expended on the buildings. The latter provide accommodation for 40 patients.

The University of Jena, which has hitherto only allowed women to matriculate in the department of physiological science, has thrown open its doors to women students in all departments, including that of medicine.

After being for some considerable time in a state of quiescence, after the passing of the plans, a decided move has been made towards the erection of the new infirmary for Alnwick, which it is estimated will cost £6,200, exclusive of furnishings and apparatus.

Post-graduate vacation courses in medicine, in connection with the University and Royal Colleges, will be held in Edinburgh during the month of September. The first lecture will be given on September 2. Full particulars may be obtained from the Secretary, University New Buildings, Edinburgh.

A skilfully organised bazaar, held in the Theatre Royal at Deal in aid of the funds of the local hospital, has realised a sum of nearly £400. One of the features of the bazaar was the model cookery book, edited by Miss McCall and containing a series of admirable recipes contributed by ladies of the district and their friends. A sum of about £25 was realised by its sale.

Principal Donald MacAlister, M.D., LL.D., D.C.L., will preside at a dinner of the Glasgow University Club, London, to be given in the Trocadero Restaurant, Piccadilly Circus, W., on Friday, May 31, at 7.15 for 7.30 p.m. It is anticipated that the Right Hon. Lord Kelvin, O.M., K.C.V.O., the President of the Club, will be present. Applications for tickets to be addressed to the Honorary Secretaries, 63 Harley Street, W.

The Albany Guild for the Care of the Sick is a model institution having for its objects "the care of the sick poor in their homes, including the employment of trained nurses, the instruction in home nursing and care of the sick, the preparation of diet for the sick, and instruction in the laws of health, morals, and wholesome living." Its annual report makes interesting reading, and it is instructive to find, by reading it, how much good work the Guild has been able to achieve with the comparatively limited means at its disposal.

